# Macropinocytosis and autophagy crosstalk in nutrient scavenging

**DOI:** 10.1098/rstb.2018.0154

**Published:** 2018-12-17

**Authors:** Oliver Florey, Michael Overholtzer

**Affiliations:** 1 Signalling Programme, Babraham Institute, Cambridge CB22 3AT, UK; 2 Cell Biology Program, Memorial Sloan Kettering Cancer Center, New York, NY 10065, USA

**Keywords:** macropinocytosis, autophagy, entosis, phagocytosis

## Abstract

Adaptive strategies used by cells to scavenge and recycle essential nutrients are important for survival in nutrient-depleted environments such as cancer tissues. Autophagy and macropinocytosis are two major mechanisms that promote nutrient recycling and scavenging, which share considerable, yet poorly understood, cross-regulation. Here we review recent findings that connect these starvation response mechanisms and discuss the implications of their crosstalk.

This article is part of the Theo Murphy meeting issue ‘Macropinocytosis’.

## 1. Introduction

**Figure 1.**
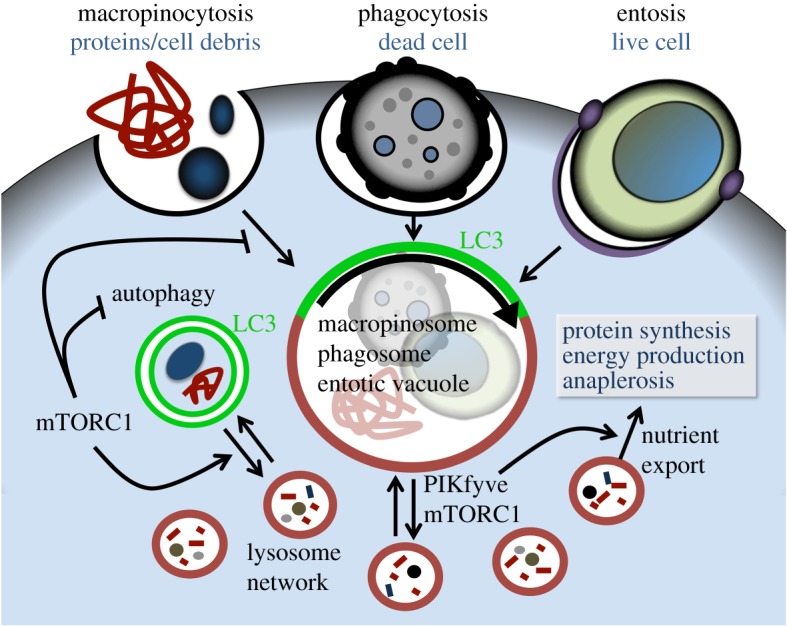
Signalling network of nutrient scavenging and recycling pathways. Cells can scavenge extracellular nutrients including proteins and cell debris through macropinocytosis, whole dead or dying cells through phagocytosis, or whole live cells through entosis. Scavenged substrates are contained within large vesicles (macropinosomes, phagosomes or entotic vacuoles) that undergo maturation involving non-canonical autophagy, or LC3 lipidation, and lysosome fusion. Degraded substrates and metabolites are redistributed into the lysosome network by PIKfyve and mTORC1-dependent fission. PIKfyve also controls the utilization of scavenged amino acids in protein synthesis that requires cytosolic export. Macropinocytic flux through lysosomal degradation is inhibited by signalling from amino acids and mTORC1. mTORC1 also inhibits the initiation of autophagy that controls the recycling of intracellular substrates, following sequestration into double-membrane autophagosomes formed through the lipidation of LC3. Following the fusion of lysosomes and autophagosomes, mTORC1 facilitates the fission of the lysosomal membrane, through autophagic lysosome reformation (ALR), to reform the lysosome network.

Cancer tissues are often starved due to a lack of sufficient vasculature to deliver glucose, amino acids, oxygen and other important nutrients [1,2]. The cell starvation that results compromises cell viability by limiting substrates needed for energy production and protein synthesis, and by initiating signalling that promotes the execution of regulated forms of cell death such as apoptosis [3–5]. One mechanism that prolongs cell viability under low nutrient and energy states is autophagy, a regulated form of self-cannibalism that recycles intracellular substrates, including proteins, protein complexes, lipids, ions and also whole organelles, through the lysosome by delivery inside specialized vesicles called autophagosomes (figure 1) [4,6]. Macromolecule digestion recycles key metabolites to allow cells to generate energy and maintain protein synthesis [4]. Autophagy is therefore one important adaptive strategy that is used by starving cells to survive periods of nutrient deprivation. In cancers, autophagy promotes disease progression in part by sustaining the viability of cancer cells in nutrient-deprived tumour microenvironments [7].

While autophagy can prolong the viability of individual cells, this process consumes intracellular substrates, and therefore the catabolism of recycled components results in cell shrinkage [5]. Proliferation that ultimately fuels cancer progression requires instead the accumulation of biomass—a doubling of the total protein, lipid and nucleotide content and even whole organelles like mitochondria—to generate all of the components needed to generate two cells from one. Cell growth in nutrient-depleted environments therefore requires different strategies to allow for the scavenging of extracellular substrates that remain abundant when nutrients such as amino acids and glucose delivered from blood are lacking. It has become clear that endocytic processes such as macropinocytosis are key strategies that allow cells to respond to starvation by scavenging bulk extracellular substrates, including proteins in the cancer microenvironment (figure 1) [8,9]. Lysosomal digestion of scavenged proteins and other macromolecules can help to fuel cell growth under conditions of starvation for conventional nutrients.

While autophagy and macropinocytosis are separate strategies, they are also collaborative in a general sense, as both are adaptive processes that allow cells to respond to starvation, and the prolonged viability afforded by autophagy can conceivably allow cells time to scavenge through macropinocytosis. But these processes also share much more direct crosstalk through their regulatory mechanisms. How crosstalk between macropinocytosis and autophagy is regulated, and how cross-regulation impacts nutrient homeostasis remains poorly understood and will be the subject of this review.

## 2. Cross-regulation through upstream signalling

### (a) Regulation of autophagy and macropinocytosis by amino acid signalling and mTORC1

One key regulator of both autophagy and macropinocytosis is the mechanistic target of rapamycin (mTORC1), a nutrient-sensing kinase that coordinates cell fate decisions in response to growth factors and numerous metabolites including glucose and amino acids (figure 1) [10]. When nutrients such as amino acids are plentiful, mTORC1 signalling promotes protein synthesis and cell growth and inhibits autophagy through phosphorylation and inhibition of the autophagy-initiating ULK kinase complex [11]. Amino acid signalling [12] and mTORC1 activity [13] also inhibit flux through macropinocytosis, in a ULK-independent manner, by limiting the capacity for lysosomal degradation of ingested protein (figure 1) [12–14]. When amino acids are scarce, protein synthesis is inhibited and autophagy is initiated to support cell survival, and the potential for maximal degradation of scavenged protein is established to facilitate cell growth. This form of crosstalk may allow cells to function in an opportunistic manner during starvation, as the initiation of autophagy could prolong cell survival, while increased macropinocytic flux could poise cells to respond efficiently to upstream signals, such as those from activated Ras [9], growth factor receptors and PI3K [15] and Wnt [16], that can initiate macropinocytosis. While growth factor signalling would also activate mTORC1, and thereby suppress macropinocytic flux [15], the parallel requirement of amino acids for mTORC1 activity [17], and the further regulation of macropinocytic flux by amino acids in an mTORC1-independent manner [12], would ensure that scavenging activity remains maximal when amino acids are scarce. Cell responses to starvation may also be tuned to the severity of the stress. For example, the reduction in the availability of even a single amino acid (e.g. leucine or glutamine) has been shown to increase macropinocytic flux, but may not inhibit mTORC1 activity that suppresses autophagy [12]. Total amino starvation, on the other hand, is a potent inducer of autophagy, acting through loss of mTORC1 activity and also additional signals, such as the PP2A-B55a phosphatase that activates ULK1 in a parallel manner [18]. Autophagy induction may therefore be reinforced as a cell response when starvation is more severe.

### (b) Entosis and autophagic lysosome reformation regulated by mTORC1

Another form of bulk nutrient scavenging that is receiving increasing attention is called entosis [19]. Through entosis, cells ingest whole live neighbouring cells and degrade them within enlarged endolysosomes, called entotic vacuoles, which resemble phagosomes in macrophages. While the downstream degradation stages of entosis resemble other forms of cell engulfment, the upstream process is distinct, as the ingested entotic cells play an active role in their uptake by burrowing into their hosts, in the absence of known initiators of phagocytic ingestion such as exposure of the ‘eat-me’ signal phosphatidylserine [19]. While mTORC1 signalling is not known to control entosis induction, the activity of this kinase has been shown to regulate the end stages of degradation that involve the redistribution of digested cargo from entotic vacuoles into lysosome networks, through mTORC1-dependent vesicle fission [20]. In this context, mTORC1 may not regulate nutrient efflux *per se*, but rather the potential for efflux by regulating vesicle fission and the redistribution of scavenged nutrients to the lysosome network. This mTORC1-regulated activity may be related to similar control over the end stages of autophagy, where a related mTORC1-dependent process involving membrane tubulation and vesicle fission, called autophagic lysosome reformation, or ALR, establishes new lysosomes from enlarged autophagolysosomes [21]. ALR occurs after prolonged periods of starvation when mTORC1 becomes reactivated, and mTORC1 activity is required for tubulation of the lysosomal membrane and a resulting resetting of lysosome networks to maintain prolonged autophagy flux. While the vesicles generated by ALR are not thought to contain cargo [21], those produced by membrane fission during entosis do contain scavenged material [20], and fission may be involved in promoting the efflux and utilization of scavenged metabolites (see also section ‘A link between vesicle trafficking and bulk nutrient scavenging’ below). This suggests, intriguingly, that while mTORC1 activity is an inhibitor of macropinocytic flux, it could promote nutrient scavenging through entosis.

### (c) Regulation by AMPK

While mTORC1 is active under nutrient-replete conditions, conversely the AMP-activated protein kinase complex (AMPK) remains inactive when nutrients are plentiful, and becomes activated in response to starvation due to the accumulation of AMP and ADP that bind directly to the kinase complex [22]. When induced, AMPK initiates autophagy by phosphorylating the ULK kinase complex and also suppresses mTORC1 activity by phosphorylating and inhibiting tuberous sclerosis complex 2 (TSC2), a GTPase-activating protein (GAP) that inhibits the mTORC1 activator RHEB (ras homologue enriched in brain) [11,23]. Recently, AMPK activity was also shown to promote nutrient scavenging through macropinocytosis [24], and through entosis [25], in response to low energy stress that results from glucose starvation. Stimulation of macropinocytosis by AMPK occurred through activation of the Rac1-GTPase, which controls actin polymerization and membrane ruffling, and was independent of ATG5, demonstrating regulation separable from autophagy [24].

For entosis, AMPK was found to regulate nutrient scavenging activity, curiously, in a non-cell-autonomous manner [25]. Entosis is known to involve invasive activity on the part of the ingested cells, which invade their hosts through a mechanism involving RhoA-GTPase, actin and myosin [19]. This process is competitive between cells in a population, as ‘losers’ become ingested and eliminated, sacrificing themselves and in the process supporting the growth and proliferation of winners that absorb their nutrients [26]. Remarkably, AMPK was found to be induced to the highest levels in starved cell populations within loser cells and was required for their uptake into winners [25]. Thus while AMPK can control autophagy, macropinocytosis and entosis in starved cells, on the one hand, it rescues individual cells from the effects of starvation in an autonomous manner through autophagy and macropinocytosis, while on the other it sacrifices the most energetically compromised cells to feed others in the population in the long term through entosis.

## 3. Direct cross-regulation through non-canonical autophagy or LAP

A direct form of crosstalk between autophagy and endoytic processes was discovered by the elucidation of an unconventional role of autophagy proteins in modifying macroendocytic vesicles, including phagosomes, entotic vacuoles and also macropinosomes [27]. This activity, called ‘non-canonical’ autophagy, is distinct and independent from the classical autophagy pathway as it does not depend on the formation of double-membrane autophagosome structures nor involve the degradation of intracellular cargo [28–31]. During classical autophagy, two ubiquitin-like conjugation systems direct the lipidation of cytosolic ATG8 proteins (e.g. LC3) onto the lipid phosphoethanolamine on membranes that form autophagosomes [32,33]. During ‘non-canonical’ autophagy, these conjugation systems instead target LC3 lipidation onto endocytic membranes, including macropinosomes [28,34]. Among the proteins comprising the ubiquitin-like conjugation machinery, an E3 ligase-like complex consisting of ATG16L1, ATG5 and ATG12, is responsible for determining which membrane is targeted for LC3 lipidation [33]. Key residues in the central domain of ATG16L1 are required to target LC3 to autophagosome structures [35–37]. However, these residues are not required for non-canonical autophagy and instead the C-terminal WD40 domain was found to be essential in targeting to single-membrane compartments [38]. Thus, by manipulating ATG16L1, autophagy and endolysosomal non-canonical autophagy can be distinguished as separate processes.

**Figure 2.**
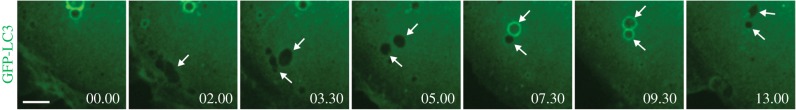
Macropinosomes undergo LC3 lipidation. Confocal time-lapse imaging is shown of KrasV12-induced macropinocytosis in MCF10A *ATG13*^−*/*−^ cells expressing green fluorescent protein (GFP)-LC3. Arrows mark newly formed macropinosomes to which GFP-LC3 is transiently recruited. Scale bar 3 µm (min:s).

Non-canonical autophagy was first identified during phagocytosis, where it was termed LC3-associated phagocytosis, or LAP [29,39–42]. Following the phagocytic engulfment of bacterial or fungal pathogens, LC3 was observed to transiently recruit to single-membrane phagosomes. This occurred independently of classical autophagy, as it did not rely on the ULK1 initiation complex, but it did require ATG5 and ATG7, which are components of the ATG8 conjugation system. The same LAP pathway was subsequently shown to function during the phagocytosis of apoptotic and necrotic cells [28,41,43,44], in both *in vitro* and *in vivo* models. Importantly, non-canonical autophagy was also shown to associate with other distinct macro-scale endocytic processes. For example, LC3 was shown to recruit to entotic vacuoles harbouring live cells formed during entosis [25,28,45] and to macropinosomes formed in a variety of cell types through either constitutive or stimuli-induced macropinocytosis [28,38] (figure 2). The non-canonical autophagy pathway commonly referred to as LAP is therefore a regulatory mechanism that affects numerous endocytic processes including macropinocytosis.

### (a) Regulation of LAP and non-canonical autophagy

The upstream signals that activate non-canonical autophagy remain to be fully elucidated but do not appear to involve regulation by mTORC1 that is an important controller of canonical autophagy. Nutrient starvation also appears unlinked to this process, as the reported instances of this non-canonical pathway involve cells cultured under nutrient-replete conditions, and the pathway appears unaffected by nutrient starvation [27]. For LAP, certain surface receptors including Toll-like receptors (TLRs) [42], Fc*γ* receptors [39], Dectin1 [40], PS receptors [41] and the *β*_2_ integrin Mac-1 [46] have been shown to be important for activating the pathway [27]. Receptor signalling leads to the generation of phosphoinositide-3-phosphate (PI3P) via the Rubicon–Vps34 complex [31]. Increased PI3P is required for efficient activation of NADPH oxidase and generation of reactive oxygen species (ROS), which are required for LAP [31,39]. However, it is not known whether these receptors and ROS production are important for all incidences of non-canonical autophagy such as macropinocytosis or entosis. Indeed, it is unclear what receptors may be involved during macropinocytosis as this is often thought of as a receptor-independent process. Interestingly, during macropinocytosis, not all macropinosomes appear to be targeted by LC3, even in the same cell. Monitoring constitutive macropinocytosis in mouse embryonic fibroblasts (MEFs) by fluorescent time-lapse imaging showed that only 50% of macropinosomes recruited LC3 [28]. Why some macropinosomes are targeted and others not is unknown. It may potentially relate to the size or fate of macropinosomes, whether degradative or recycling in nature. It is likely that multiple signals exist associated with specific engulfment programmes, but which culminate and converge on a more universal regulatory mechanism of non-canonical autophagy. One such regulatory candidate is the function and activity of the V-ATPase. Inhibition of V-ATPase using Bafilomycin A was seen to inhibit LC3 recruitment during a range of non-canonical autophagy-associated processes including LAP, while not affecting upstream phagosome maturation events [34].

### (b) Functions of non-canonical autophagy

How the non-canonical autophagy modification of endolysosomal membranes functions during macroendocytic processes is not fully understood. A number of studies have proposed that the recruitment of ATG8 proteins modulates the maturation of macroendocytic vesicles. During LAP and entosis, LC3 was shown to target to phagosomes at a specific stage of their maturation. Using fluorescent time-lapse microscopy, LC3 was observed to recruit after the generation of PI3P, recruitment of Rab5 and before the recruitment of Rab7, LAMP1 and the acidification of the phagosome [28,42]. This suggests that non-canonical autophagy might regulate the transition from an early to late phagosomal compartment. In LAP-deficient macrophages, for example, lacking ATG5 or ATG7, there was indeed an observed defect in the recruitment of lysosomes and acidification of phagosomes containing pathogens [31,40–42,46], which corresponded to a defect in pathogen killing and clearance. A similar LAP-dependent role for lysosome fusion and degradation of phagocytic cargo was also observed during phagocytosis of apoptotic cells [28,31,41,43,44] and in the clearance of shed photoreceptor outer segments (POS) by retinal epithelial cells. Importantly, LAP-dependent processing of POS could support the recycling of retinoids necessary to maintain the visual cycle in mice [30]. Similarly, during entosis, the siRNA-mediated knockdown of ATG5 or ATG7 reduced LC3 recruitment to entotic vacuoles and the killing of internalized cells [25,28,47], thus implicating this pathway in the entotic scavenging of nutrients. The presentation of exogenous antigen on major histocompatibility complex class II by professional antigen-presenting cells represents another engulfment-associated process where there is now strong evidence showing a role for non-canonical autophagy [38,40,48].

These studies all point to a potential role for non-canonical autophagy in promoting lysosome fusion. ATG8 proteins could function in this context by binding to any of a host of known interacting proteins [49] to control endosome maturation. This function may also be context-specific, as the presence of LC3 on phagosome membranes in human dendritic cells was shown, conversely, to inhibit fusion with lysosomes [48]. Another study found no defect in lysosome fusion or phagosome acidification in murine bone marrow-derived macrophage in the absence of LAP [50]. Therefore, while evidence suggests that non-canonical autophagy modulates the fusion of lysosomes and phagosomes, there may be context-dependent, species or cell type-specific differences. Emerging work also suggests that non-canonical autophagy may modulate endosomal signalling involving TLRs, B cell receptor (BCR) and integrins [46,51–54]. This raises the concept that ATG8 decoration alters the signalling potential of phagosomes, macropinosomes or endosomes, possibly through ATG8-dependent recruitment of adaptor proteins and modulation of vesicle trafficking. How LAP-like non-canonical autophagy modulates macropinocytosis is an exciting new area of research for this important process.

## 4. The products of nutrient recycling and scavenging

### (a) Autophagy and macropinocytosis

While the initiating stages of autophagy and macropinocytosis are well studied, by contrast, the downstream stages that regulate the recovery of key nutrients from lysosomes remain less well understood. What is recycled or scavenged? Early studies in yeast demonstrated that autophagy-deficient cells, or cells with inhibited vacuolar degradation, had reduced levels of amino acids and lowered levels of protein synthesis during nitrogen starvation (a potent inducer of autophagy in yeast), demonstrating that amino acids are one key nutrient that is recycled [55]. Amino acids are also important products of autophagic recycling in mammalian cells and in mice, where autophagy deficiency in response to starvation after birth is associated with decreased tissue and plasma amino acid levels [56], and in adulthood with decreased levels of plasma arginine. Starved autophagy-deficient adult mice die as a result of acute hypoglycaemia, an effect potentially linked to a lack of amino acid recycling to support gluconeogenesis in the liver [57]. In pre-implantation development, amino acids are also an important recycled substrate derived from the autophagic turnover of maternal proteins, which supports the synthesis of new zygotic proteins in the oocyte [58].

Macropinocytic scavenging is also targeted toward amino acids. Albumin is one key scavenged protein that has been shown to rescue cancer cells from amino acid starvation and to contribute to the free amino acid pool in pancreatic tumours [59,60]. As amino acids are orders of magnitude more abundant in plasma in protein as compared to free form [60], the macropinocytic scavenging of albumin, the most abundant plasma protein, is one important strategy that promotes the growth of cancer cells when blood is not in abundant supply. Non-cancer cells such as macrophages can also use macropinocytosis to feed [61], suggesting that scavenging activity could more generally contribute to provide amino acids to cancer tissues. In addition to albumin, the ingestion of proteins from the extracellular matrix, including fibronectin [59] and laminin [62] can also contribute to the free amino pool in cells [62], suggesting that long-lived matrix proteins, including those deposited by stromal cells, may be important sources of amino acids in cancer tissues. Matrix proteins are likely ingested by receptor-mediated endocytosis [62], a process that, like macropinocytic flux, is upregulated under amino acid starvation conditions when matrix secretion by stromal cells is also increased [62]. Interestingly, a percentage of ingested albumin may also enter some cancer cells through a receptor-mediated endocytic route rather than through macropinocytosis [24].

In addition to amino acids, other key metabolites such as ions [63], lipids and nucleosides are recycled or scavenged to support the survival or growth of starved cells. Nucleosides have emerged as a key recycled substrate that supports the survival of starved cells. Amino acid starvation or mTORC1 inhibition induces the autophagic turnover of ribosomes [64], through the ribophagy receptor NUFIP1-dependent binding of ribosomal large subunits to LC3. While ribophagy leads to the recycling of amino acids [64], the lysosomal degradation of ribosomal RNA also generates nucleosides that are exported and used as energy-generating substrates to support cell survival or nucleic acid synthesis. Nucleosides generated as products of autophagy were also previously shown to promote the survival of Ras-mutant lung cancer cells in response to starvation [65].

Scavenging activity can also be directed toward lipids. In particular, monounsaturated fatty acids have been shown to be harvested by Ras-mutant cancer cells directly from serum, in the form of lysophospholipids, which supports proliferation [66]. Lysophospholipids could enter cells through macropinocytosis, although the specific mechanism underlying their uptake in cancers is unknown. Fatty acids can also enter cells through macropinocytosis by binding to albumin in the serum. Through autophagy, fatty acids are also important metabolites for recycling, through either whole organelle turnover [67] or lipid droplet degradation (called ‘lipophagy’), which are upregulated in starved cells to promote the recycling of fatty acids that are used as an energy source in mitochondrial metabolism through β-oxidation [67,68]. Thus, numerous metabolites, in addition to amino acids, are recovered through autophagic recycling or macropinocytic scavenging mechanisms to support the survival or proliferation of starved cells.

### (b) Bulk cell scavenging

In addition to individual classes of macromolecules such as extracellular proteins or lipids, bulk substrates such as whole cells or cell fragments can also be scavenged, and these may supply large quantities of nutrients that are well suited to support cell growth. Necrotic debris was recently shown to be scavenged by cancer cells through macropinocytosis in a manner that could support growth and proliferation. The ingestion of necrotic debris restored the lipid droplet content of starved prostate cancer cells, providing evidence that scavenging of lipids from necrotic cell fragments can contribute to lipid storage [24]. Apoptotic cells have similarly been shown to act as a nutrient source for macrophages to support survival and proliferation, and in this context amino acids from phagocytosed corpses were shown to be scavenged and used in protein synthesis [20]. Similarly, the ingestion of whole cells through entosis, or through related mechanisms such as cell cannibalism [69], can support cancer cell survival and proliferation, suggesting that the scavenging of whole cells or cell fragments can support proliferative potential, presumably by supplying numerous nutrients in bulk, from amino acids and lipids, to nucleosides, ions cofactors and potentially sugars.

### (c) A link between vesicle trafficking and bulk nutrient scavenging

Bulk nutrient scavenging of cells involves the formation of macro-scale vesicles with sizes ranging from 5 to up to 15 µm in diameter [28]. Macropinosomes can also be large and range in diameter from one to several microns. While sequential maturation events involving RAB GTPases and LC3 lipidation participate in regulating the fusion of lysosomes to these vesicles, the scavenged macromolecules undergoing degradation are contained within large vesicles whose membranes must undergo fission in order to, ultimately, shrink in size and disappear. mTORC1 activity was the first identified regulator of this late stage of vesicle shrinkage, controlling the fission of apoptotic cell phagosomes in macrophages and entotic vacuoles in epithelial cells [20]. Now a recent study identified an additional, parallel regulator of this end stage: the lipid kinase PIKfyve, an enzyme that catalyses the conversion of phosphinositol-3-phosphate (PI-3-P) on endosomal membranes to PI-3,5-P2 [14,70]. PIKfyve activity, like mTORC1, regulates fission of the endolysosmal vesicles harbouring ingested extracellular substrates, in a manner that redistributes ingested material from phagosomes, entotic vacuoles and macropinosomes, into the lysosome network of engulfing cells [14]. When PIKfyve activity is blocked, engulfed contents are no longer redistributed and utilization of the scavenged amino acids in protein synthesis was also inhibited, suggesting a link between vesicle fission and the cytosolic export of nutrients. PIKfyve activity was shown to be required for amino acid scavenging from ingested apoptotic cells in macrophages, and also for the utilization of ingested albumin to support the proliferation of amino-acid-starved cancer cells [14]. These findings demonstrate that distinct scavenging pathways share downstream regulation through PIKfyve, which controls the cytosolic nutrient export and also shrinkage of the large endocytic vesicles that harbour ingested nutrient sources.

## 5. Conclusion

Here we have reviewed recent findings that link adaptive mechanisms of nutrient recycling through autophagy and nutrient scavenging through macropinocytosis, phagocytosis and entosis. Crosstalk between these various mechanisms is controlled by upstream signalling pathways that regulate cell responses to nutrient starvation, by direct cross-regulation mediated by autophagy pathway proteins and by downstream regulators of nutrient efflux. Understanding how these interconnected pathways are cross-regulated may ultimately open new avenues to further our understanding of the disease. For some aspects of the crosstalk discussed here, implications for cancer therapy seem relatively straightforward. For example, mTOR inhibitors are in clinical trials for the treatment of various cancers but have thus far shown, in most cases, limited efficacy [71–73]. While the mechanistic underpinnings of this are likely complex, enhanced macropinocytic scavenging activity linked to increased lysosomal flux resulting from mTOR inhibition could be one underlying factor [13]. mTOR inhibition has indeed been shown to enhance cell proliferation in pancreatic tumour regions that are poorly vascularized, while inhibiting proliferation at tumour margins [13]. It is conceivable that inhibiting the utilization of scavenged protein, for example, by blocking the cytosolic export of amino acids that require PIKfyve activity, could enhance the anti-cancer effects of mTOR inhibition. In culture models, the pharmacological inhibition of PIKfyve activity largely blocks the pro-proliferative effects of mTOR inhibition, when the growth of various cancer cells is supported by albumin scavenging [14]. PIKfyve inhibition has also been shown to limit the growth of multiple cancer types in mice, suggesting that PIKfyve is a promising therapeutic target whose inhibition may block lysosomal nutrient recovery [74,75].

While the discovery of LAP and related pathways involving endolysosmal LC3 lipidation, or ‘non-canonical autophagy’, demonstrated clear evidence of crosstalk between autophagy pathway proteins and endocytic scavenging mechanisms, the upstream regulation of these pathways appears to be largely distinct, and no clear effects of canonical autophagy engagement on LAP-related pathways are known. Strategies to inhibit canonical autophagy for therapeutic benefit, involving, for example, the targeting of upstream signalling modules such as the ULK kinase complex, or mTORC1, are not predicted to affect the function of autophagy proteins in endocytic trafficking, as LC3 lipidation in this context appears independent from these known upstream signalling regulators of autophagy. The molecular mechanism(s) underlying control over macroendocytic vesicle maturation by the lipidation of ATG8 still remain unclear. While LC3 lipidation appears to be required for efficient lysosome fusion and/or degradative capacity in multiple systems, it is unclear whether macropinocytic scavenging of an extracellular protein involves such a role [13,24], despite the lipidation of LC3 onto macropinosomes (figure 2) [28]. In further studies, it will be important to uncover how LC3 lipidation affects macropinosome maturation. Intriguingly, the treatment of cells with numerous lysosomotropic drugs, including chloroquine [34], has been shown to promote endolysosmal LC3 lipidation, suggesting the possibility that treatment with lysosome inhibitors in a clinical setting could block nutrient recycling and scavenging activity, while at the same time promoting LAP-like LC3 lipidation. Elucidation of the full repertoire of effects of this form of LC3 lipidation, across different cell types, on critical cellular activities such as endocytic trafficking, nutrient sensing, lysosomal degradation and cytokine secretion, remains an important area for future study. Recent genetic approaches to distinguish this form of non-canonical autophagy from canonical autophagy should facilitate these efforts [31,38].

## Data accessibility

This article has no additional data.
